# Diagnosis and treatment challenges in a patient with splenic tumor and chronic viral hepatitis

**Published:** 2015

**Authors:** SC Firulescu, DL Săndulescu, ŞC Dinescu, IA Gheonea, RM Purcarea, SM Săndulescu

**Affiliations:** * Department of Internal Medicine, Emergency County Hospital Craiova, Romania; ** Research Center of Gastroenterology and Hepatology, University of Medicine and Pharmacy Craiova, Romania; *** Radiology and Imaging Department, University of Medicine and Pharmacy Craiova, Romania; **** ”Carol Davila” University of Medicine and Pharmacy, Bucharest, Romania; "Dr. Carol Davila" Clinical Hospital of Nephrology, Bucharest, Romania; ***** Surgery Department, Emergency County Hospital Craiova, Romania

**Keywords:** non-Hodgkin’s lymphoma, delta hepatitis, contrast enhanced ultrasound, splenic tumor

## Abstract

Non-Hodgkin lymphoma is a cancer of the lymphatic tissue located in various parts of the body: lymph nodes, spleen, thymus, adenoids, tonsils, and bone marrow. The disease occurs mainly in adults, with a higher incidence within the age range of 45 to 60 years. We present a clinical case of non-Hodgkin lymphoma diagnosed in a patient with chronic viral hepatitis B and D. The particularity of this case consists in the diagnosis of primitive spleen lymphoma, described in less than 1% of the cases, and also the difficult antiviral therapy recommendation for the liver disease, given the associated co-morbidity.

**Abbreviations:** NHL = Non-Hodgkin lymphoma, HDV = Hepatitis delta virus, HCV = Hepatitis C virus, HBV = Hepatitis B virus, CT = Computerized tomography, CEUS = Contrast enhanced ultrasonography, CHOP = cyclophosphamide, doxorubicin, vincristine, prednisone, R-CHOP = cyclophosphamide, doxorubicin, vincristine, prednisone and rituximab

## Introduction

Lymphomas are malignancies of the lymphoid cells located in various tissues. The two major forms of lymphoma are Hodgkin lymphoma and non-Hodgkin lymphoma (NHL). Although the spleen is involved in 30-40% of the cases of systemic lymphoma, primary spleen lymphoma is a rare occurrence, less than 1% of the cases [**1**].

Hepatitis delta virus (HDV) is a defective RNA virus, the unfolding of its replication and transmission processes being dependent on the presence of the hepatitis B surface (HBs) antigen. Thus, hepatitis delta can occur as either a co-infection or a super-infection in patients with chronic hepatitis B (CHB). In Southern Europe, hepatitis Delta can be defined as a highly endemic disease, with more than 20% of HBs antigen positive patients testing positive for anti-HDV antibodies [**2**]. Although epidemiological studies in Europe are showing a decrease in the prevalence of chronic viral hepatitis Delta (CHD), reports in Romania show an increased prevalence of the HDV genotype 1, being identified in 20,4 % of the cases [**2**].

According to literature published in Romania, no differences between the prevalence rates for chronic hepatitis B, C, D in patients with lymphoproliferative disorders were observed, these cases showing low levels of viral replication [**3**]. The reported prevalence of hepatitis B and D viral infection in patients with NHL is 2.43% [**4**].

Positive diagnosis of splenic NHL is based on the identification of the tumor mass, through imaging techniques, followed by confirmatory histopathological examination. Diagnosis of HBV/ HDV co-infection relies on the detection of the specific antigens or antibodies, by means of immunological testing.

## Case presentation

We present the case of a 37-year-old female patient, coming from rural area, who presented on admission with pain in the left upper quadrant, fever with insidious onset, lasting for approximately 3 weeks. Also, the patient mentioned a history of chronic hepatitis B infection, diagnosed at the age of 9.

Physical examination revealed diffuse abdominal pain, more pronounced in the left upper quadrant and splenomegaly grade 2.

Blood tests showed normal blood counts, presence of a moderate inflammatory syndrome with ESR (erythrocyte sedimentation rate) – 52mm/ 1h (N <20mm), normal liver and kidney function. Virological tests showed negative anti-HIV antibodies, positive HBs antigen, negative HBe antigen, negative anti-Hbe antibodies, and negative anti-HCV antibodies. Viral load assessment revealed the following results: HBV DNA - 306 IU/ ml, HDV RNA - 2.759.000 IU/ ml. Based on the immunological profile and viral load levels, the patient was diagnosed with HBV/ HDV co-infection (with suppression of HBV replication). For the non-invasive diagnosis of liver fibrosis, a Transient Elastography was performed (FibroScan® device -EchoSens, Paris, France), which measured a Metavir score F2, evocative for significant liver fibrosis.

**Fig. 1:**
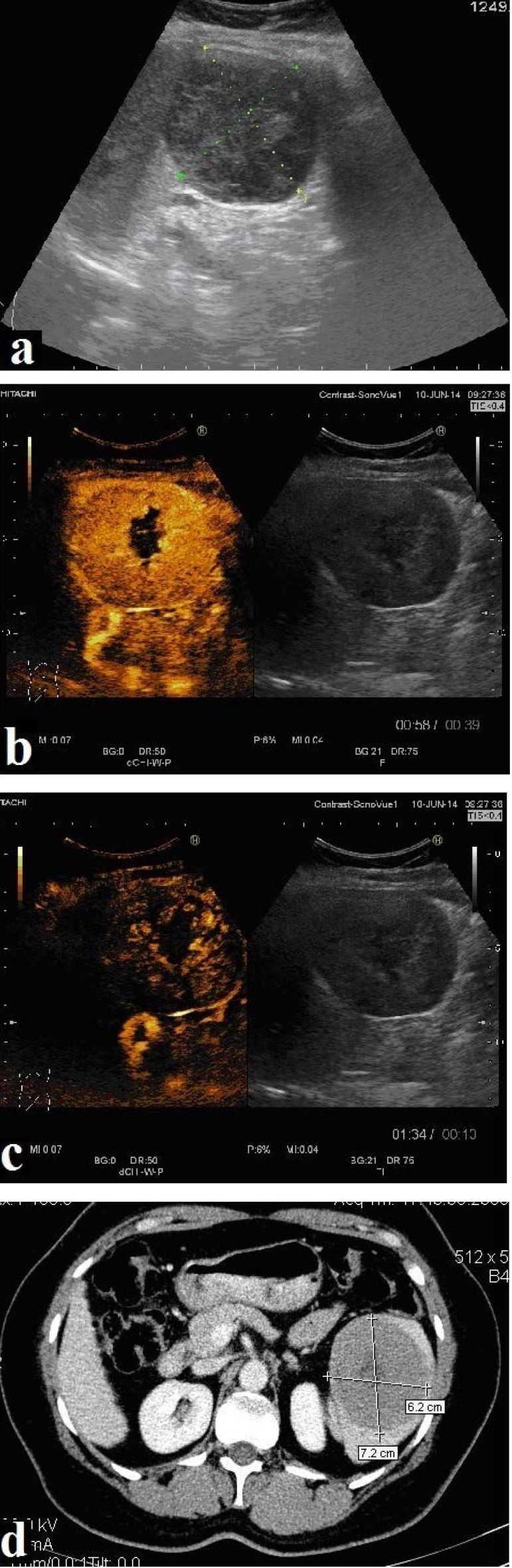
Abdominal ultrasound: splenic mass 7.4/ 6.8 cm, round-oval in diameter, located at the inferior pole of spleen, with an intense inhomogeneous structure (Fig. 1a). Contrast enhanced ultrasonography in the arterial phase shows a rapid uptake of the contrast agent in the splenic mass, 10 seconds after injection, with a central area of necrosis (Fig. 1b). Rapid and complete washout is revealed in the late phase (Fig. 1c). Computer tomography shows heterogeneous mass located at the lower pole of spleen spontaneously isodense, moderately iodophilic, with necrotic areas and minimal extracapsular extension (Fig. 1d)

The abdominal ultrasound examination detected a well-defined, round-ovalar splenic mass, of 7.4/ 6.8 cm in diameter, located at the inferior pole of the spleen, with an intense inhomogeneous structure (**Fig. 1a**).

Contrast enhanced ultrasonography showed a rapid uptake of the contrast agent in the splenic tumor, 10 seconds after injection, with a central area of necrosis, with rapid and complete washout in the late phase, 60 seconds after injection (**Fig. 1b**,**c**).

CT scan described an enlarged spleen with the presence of heterogeneous round-ovalar, relatively well-defined mass, arising from the lower pole, spontaneously isodense, moderately iodophilic, with necrotic areas and minimal extracapsular extension (**Fig. 1d**). 

**Fig. 2:**
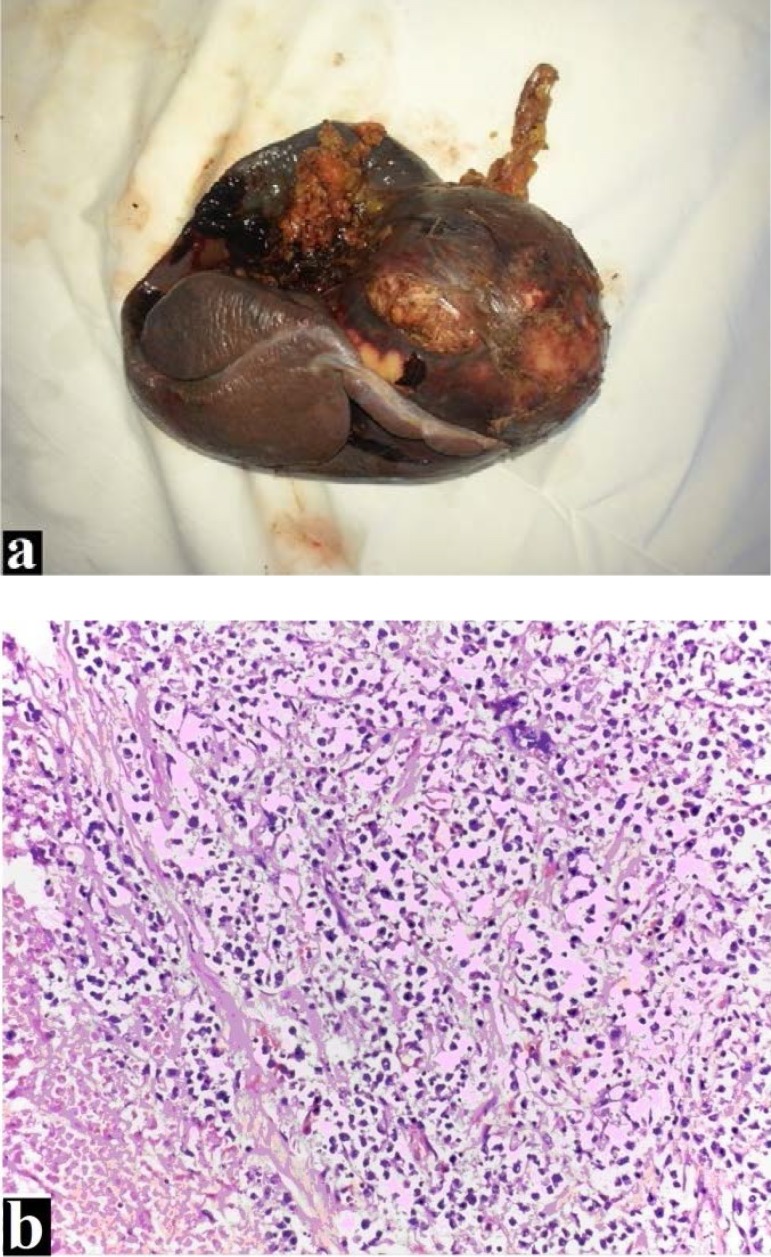
Splenic mass after splenic resection. Macroscopic view (Fig. 2a) and histopathologic analysis (HE stain, 20X) (Fig. 2b)

Based on the imaging findings, a diagnosis of malignant tumor was established. In order to determine the exact nature of the splenic tumor, surgical splenectomy coupled with histopathological evaluation was performed. Microscopic examination revealed splenic parenchyma with architectural distortion and presence of large and medium-size malignant cell proliferation, suggestive large B cell lymphoma with Burkitt-like areas (**Fig. 2a**,**b**). 

The patient was directed to the hematology clinic, in order to initiate the CHOP cure (cyclophosphamide, doxorubicin, vincristine, and prednisone). After one CHOP cure, the laboratory work-up showed notably increased cytolysis enzymes, thus imposing the treatment discontinuation. Given the patient’s refusal to follow a pegylated interferon line of therapy and the early-stage liver disease (fibrosis stage F2), it was decided to postpone the antiviral therapy until hematologic remission.

## Discussions

The ultrasound aspect of splenic lymphoma lesion varies from diffuse miliary to micronodular infiltrates or macronodular appearance. CEUS can differentiate between malignant and benign lesions, based on the dynamic absorption and elimination (washout) pattern of the contrast agent, during the arterial and late phase. CEUS reaches a good specificity in the differentiation of benign from malignant splenic lesions, as hypo-enhancement in the parenchymal phase is predictive of malignancy in 87% of the cases [**5**]. In the present case, CEUS and CT features advocated for a malignant tumor, without being able to specify its type, surgery, and histopathological examination being required. 

In this clinical case, the diagnosis of a primary splenic lymphoma in a patient with chronic hepatitis B and D created a significant challenge in choosing the appropriate antiviral treatment. According to the clinical trial data published so far, pegylated interferon alpha represents the only currently available antiviral therapy for chronic Hepatitis B+D. Of note, HDV is reported to be the dominant virus in most cases of HBV/ HDV co-infection, with higher HDV viral load levels, compared to the replication levels of the other viruses [**6**]. Response rates reported in CHB+D patients treated with pegylated interferon alpha varied between 17-43%, following a treatment period of 48 weeks or 72 weeks [**2**,**7**]. Studies published to date included a relatively small number of CHD patients with non-hematological comorbidities. A recently published study from Romania, including 50 patients with CHB+D and compensated liver cirrhosis treated with pegylated interferon alpha for 48 weeks, demonstrated a 12% rate of sustained virologic response [**8**].

In this case, disease progression, in the absence of antiviral treatment, will be determined by both aggressiveness of the lymphoma and evolution of liver disease. Standard NHL therapy is represented by CHOP. An important consideration is viral hepatitis infection reactivation as a result of chemotherapy [**4**]. Adding rituximab to standard CHOP therapy can also lead to increased viral replication, occurrence of acute fulminant hepatitis and subsequent death [**9**]. In our case, the introduction of antiviral therapy with pegylated interferon was possible only after developing a complete response to NHL therapy.

Scientific data available so far, pointed out those hepatitis viruses are not only hepatotropic, but also lymphotropic, with evidence suggesting they can induce lymphoproliferative malignancies [**10**]. This suggests a potential causal relationship between viral hepatitis infection and NHL. It is important to continue our research efforts to better understand the mechanisms by which hepatitis viruses can induce lymphoproliferative disorders, particularly NHL, in order to take the best therapeutic approach and be able to slow down or stop disease progression.

## Conclusions

Diagnosis of primitive spleen lymphoma in patients with NHL is rare, less than 1% of the cases. Contrast enhanced ultrasound and CT scan with contrast agents can guide diagnosis but histopathological examination is often necessary. Co-existence of chronic viral hepatitis B and D with primitive spleen lymphoma can be demanding in terms of therapeutic approach. Finding the ideal timing for antiviral therapy initiation is challenging, given the associated lymphoproliferative syndrome.

**Acknowledgments**

This paper was published under the frame of European Social Found, Human Resources Development Operational Program 2007–2013, project no. POSDRU/159/1.5/133377.

**Disclosures**

None.
